# Dispersion Homogeneity of Silicon Anode Slurries with Various Binders for Li-Ion Battery Anode Coating

**DOI:** 10.3390/polym15051152

**Published:** 2023-02-24

**Authors:** Bogyoung Kim, Yeeun Song, Byungwook Youn, Doojin Lee

**Affiliations:** Department of Polymer Science and Engineering, Chonnam National University, Gwangju 61186, Republic of Korea

**Keywords:** polymer binders, silicon anode slurries, rheology, slurry coating, lithium-ion battery

## Abstract

We aimed to determine the relationship between surface chemistry and the rheological properties of silicon anode slurries in lithium-ion batteries. To accomplish this, we investigated the use of various binders such as PAA, CMC/SBR, and chitosan as a means to control particle aggregation and improve the flowability and homogeneity of the slurry. Additionally, we utilized zeta potential analysis to examine the electrostatic stability of the silicon particles in the presence of different binders, and the results indicated that the conformations of the binders on the silicon particles can be influenced by both neutralization and the pH conditions. Furthermore, we found that the zeta potential values served as a useful metric for evaluating binder adsorption and particle dispersion in the solution. We also conducted three-interval thixotropic tests (3ITTs) to examine the structural deformation and recovery characteristics of the slurry, and the results demonstrated that these properties vary depending on the strain intervals, pH conditions, and chosen binder. Overall, this study emphasized the importance of taking into account surface chemistry, neutralization, and pH conditions when assessing the rheological properties of the slurry and coating quality for lithium-ion batteries.

## 1. Introduction

Silicon (Si) has emerged as a promising material for lithium-ion battery anodes due to its high energy density and capacity [1,2]. Anode slurries composed of silicon particles and a binder have the potential to significantly improve the performance of these batteries, which are widely used in portable electronics, electric vehicles, and renewable energy storage systems [3]. However, silicon is susceptible to cracking and pulverization during the battery charging and discharging cycles, which can severely limit the anode’s performance [4,5]. This can lead to poor electron transport, causing a rapid decline in capacity, as the particles lose contact with the current collector and the contact area between silicon and the conductive material is reduced. Therefore, research efforts are currently focused on enhancing the stability and cycling performance of silicon-based anodes.

The utilization of binders in the composition of lithium-ion battery electrodes is essential, as they are responsible for maintaining the structural integrity of the active materials and promoting cohesiveness within the electrode framework. As a critical component of the electrode, binders provide stability and support to the active materials, significantly contributing to the proper functioning of the battery. There are various types of binders that can be utilized in the production of lithium-ion batteries with silicon-based electrodes. In particular, water-soluble binders that are compatible with silicon active materials have several advantages such as biodegradability, a reduced risk of overheating-induced fires, and good film-forming properties on the electrode [6,7]. It is crucial to note that the adsorption of water-soluble binders on lithium-ion battery electrodes is contingent on several critical factors. These include the charge density of the particles, the size and shape of the particles, the surface properties of the particles, and the molecular structure of the binders [7,8]. Additionally, the binders must be able to maintain connectivity between the active electrode materials and the current collector, by forming a strong bond between them, to ensure efficient current flow through the electrode. The interaction of binders with silicon active materials is based on the silanol functionalities on the oxide surface of the silicon particles [9,10,11]. Hence, the binding strength between the binder and the particles will depend on the specific properties of both the binder and the particles and can be optimized by carefully controlling these factors. The ideal pH for lithium-ion battery silicon anodes varies based on the specific materials and electrolytes used in the battery [11,12]. In general, a pH between 3 and 5 is considered optimal for lithium-ion battery electrolytes, as the performance of these batteries tends to be best when the electrolyte is slightly acidic [10,11]. However, it is important to note that the specific pH range that is optimal for a particular lithium-ion battery may vary depending on the specific materials and design of the battery. Some research suggests that lithium-ion batteries with silicon anodes may perform best at slightly higher pH values, which can help to improve the stability of the electrolyte and inhibit lithium dendrite growth, while also reducing the degradation of the silicon anode [13,14]. Thus, it is essential to investigate the binding effect and adsorption properties of various water-soluble binders on the silicon particles, as the binder adsorption significantly affects the rheological properties of silicon anode slurries and subsequently the coating thickness and homogeneity on an electrode [15,16].

Motivated by this, we investigated the binding effect of various water-soluble binders with silicon nanoparticles to explore the deformation and recovery characteristics of silicon anode slurries, which have a significant impact on the coating efficiency of the slurries on the electrode. In this study, we examined the rheological properties of the silicon anode slurries to identify the structural networks and to plot useful structural deformation parameters. We investigated the structural deformation and recovery characteristics of the silicon anode slurries using the structural deformation parameters in a phase diagram to identify the criteria that can promote efficient slurry coating on the electrode. This allowed us to comprehend the rheological properties of the silicon anode slurries with various water-soluble binders and their coating efficiency.

## 2. Experiments

### 2.1. Materials

A commercial silicon powder (US Research Nanomaterials Inc., USA) with an average particle size of 30–50 nm was used as the anode active material. Commercial carbon black (CB) with a mean particle diameter of 30–50 nm (Super P, TIMCAL Graphite & Carbon Co., Belgium) was used after purification as a conductive agent. Styrene-butadiene rubber (SBR) in the form of a 50 wt% aqueous emulsion with a viscosity of 100 mPa·s at 25 ℃ was used. Sodium carboxymethyl cellulose (CMC) (Sigma Aldrich, USA) having a molecular weight of 700,000 g/mol was used with the SBR. The mixing ratio of CMC/SBR was 1:1. Chitosan (Sigma Aldrich, USA) with a molecular weight of 375,000 g/mol and poly(acrylic acid) (Sigma Aldrich, USA) with a molecular weight of 3,000,000 g/mol were used as a binder. Extra-pure-grade acetic acid glacial (Duksan Pure Chemical Co., Seoul, Republic of Korea) was used.

### 2.2. Preparation of Si Anode Slurries

A planetary centrifugal mixer (Thinky, ARE-310, Japan) was employed to prepare silicon anode slurries. The silicon active materials, carbon black conductive agents, and water-soluble binders were mixed in a 6:2:2 weight ratio. The preparation of the premixed suspensions involved blending the components using a planetary centrifugal mixer and ultrasonication. For the premixed PAA-based binder suspensions, silicon, and CB particles were mixed in DI water for 10 min. A 1:1 ratio of CMC and SBR was prepared for the premixed CMC-/SBR-based binder suspensions, mixed with DI water, and then, subjected to centrifugal mixing and ultrasonication. The premixed chitosan-based binder suspensions were prepared by adding 25 wt% chitosan into a 1 wt% acetic acid aqueous solution. All the premixed anode slurries were vigorously mixed by using a planetary centrifugal mixer for 10 min at 2000 rpm, followed by ultrasonication for 30 min. The suspensions were finally mixed with a magnetic stirrer for 12 h to prepare the silicon anode materials. The mixed silicon anode slurries for each step are shown in the Supplementary Information (Figure S1). The manufactured viscous slurries were deposited on a copper foil using a doctor blade to achieve a thickness of 100 μm. To measure the electrical potential at the silicon particle surface with various binders, silicon anode solutions containing silicon particles at 0.05 wt% and a small amount of binders were prepared.

### 2.3. Characterization of Coating Efficiency

The adhesion strength of the silicon anode slurries on the electrode was measured using a universal testing machine (UTM, MCT-2150, A&D Company, Japan). The slurry-coated electrode with dimensions of 10 mm in width and 40 mm in length was attached to 3M 810 Scotch tape, and both the electrode and Scotch tape sides were clamped and elongated to peel off the coated slurry at a constant rate of 50 mm/min. The zeta potential of the silicon anode slurries was measured using a laser Doppler method (Zetasizer Nano ZSP, Malvern Instruments, UK). To determine the morphology of the anode electrode, field-emission scanning electron microscopy (FE-SEM, Gemini 500, ZEISS, Germany) was utilized for measurement. The amount of binders adsorbed on the silicon particle surface was determined by centrifuging the silicon anode suspensions at 8000 rpm to separate the silicon particles firmly bound with the binders. After centrifuging, the silicon particles were dried overnight in an oven at 60 ${}^{\circ }$C and the total amount of binders was measured using thermogravimetric analysis (TGA, TGA2, Mettler Toledo, Switzerland).

### 2.4. Characterization of Rheological Properties

The rheological properties of the silicon anode slurries were measured using a stress-controlled rheometer (MCR 302, Anton Paar, Austria) at room temperature and with a plate–plate geometry of 50 mm in diameter. A small-amplitude oscillatory shear (SAOS) test was conducted to analyze the viscosity and structural deformation characteristics. The steady shear viscosity was measured at shear rate range of 0.01 to 100 s${}^{-1}$, and the strain amplitude sweep test was performed at a shear strain range of 0.1 to 100% at a 3 rad/s angular frequency. The silicon anode slurries within the measuring geometry were surrounded by heavy mineral oil to prevent the evaporation of water during the measurement. The thixotropic behaviors and structural transitions were analyzed by performing three-interval thixotropy tests (3ITTs) using a rheometer.

## 3. Results and Discussion

### 3.1. Particle Dispersity and Rheology of Si Anode Slurries

**Figure 1:**
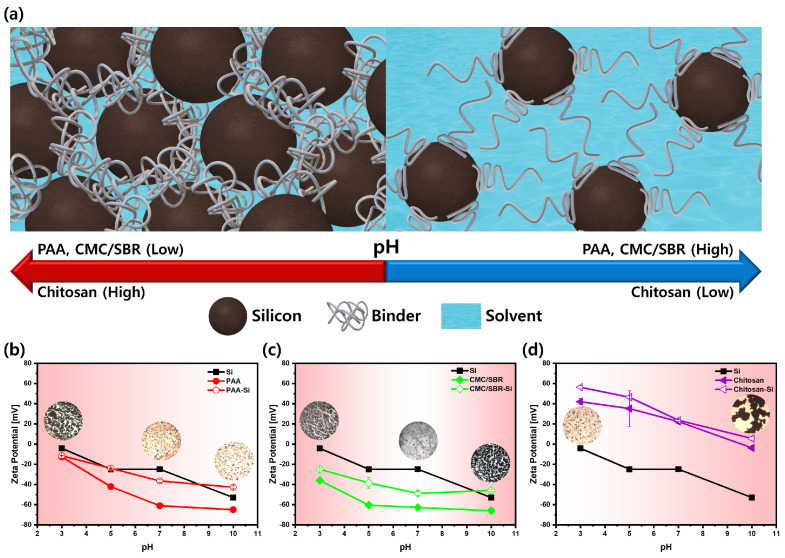
(**a**) Schematic representation of silicon anode slurries: the interaction between binders and silicon particles depending on the pH conditions. Zeta potential analysis for evaluating silicon nanoparticle stability in the solutions under various pH conditions with different binders: (**b**) PAA binder, (**c**) CMC/SBR binder, and (**d**) chitosan binder.

The surface chemistry of the elements plays a crucial role in determining the rheological properties of silicon-based anode slurries. By utilizing a combination of steric and electrostatic stabilization mechanisms, binders can effectively adhere to a surface of silicon powder and create an elastic barrier that prevents particle aggregation, thereby improving the flowability and homogeneity of the slurry [17,18,19]. The conformations of the binders on the silicon particles can be influenced by both neutralization and the pH conditions. Depending on the pH conditions, the conformation of the binders on the silicon particles can be changed, which, in turn, can affect the surface properties and ultimately how they interact with other particles (Figure 1). Binders containing carboxylate groups can form chemical bonds with Si particles through reactions between the carboxyl group of the binder and the hydroxyl group on the surface of the Si. The charge of the carboxylate groups can be further modified by altering the pH, leading to an alteration in conformation and ultimately affecting how these binders interact with other particles.

The zeta potential is a parameter that reflects the electrostatic repulsion between similarly charged particles in a colloidal suspension, providing information on the stability of the suspension and the repulsive forces between the particles. The magnitude and sign of the zeta potential can be utilized to predict the stability of the suspension and control the interaction between the particles and surfaces. Higher absolute values of the zeta potential lead to greater distances between particles and increased stability, while lower zeta potentials can result in aggregation [20]. To investigate the electrostatic stability of silicon particles in the presence of various binders, zeta potential analysis was performed (Figure 1b–d). The adsorption of binders on carbon black was excluded from the analysis as it was determined to have a negligible effect on the zeta potential change [21]. In Figure 1b–d, Si represents the silicon suspension without a binder, PAA, CMC/SBR, and chitosan are the solutions with binders only, and PAA-Si means the silicon/binder suspension. The results of the analysis indicated that, across the entire pH range, the solutions exhibited absolute surface charges ranging from 4 to 50 mV. The zeta potential values decreased with the addition of the PAA, CMC, SBR, and CMC/SBR binders, with the exception of the chitosan binder [22]. The negative potentials observed in the PAA and CMC solutions can be attributed to the dissociation of carboxyl group (-COOH), while the negative charge in the SBR is caused by an anionic surfactant during the fabrication of SBR latex mixtures [22]. In contrast, chitosan displays positively charged solutions with zeta potentials ranging from 0 to 60 mV [23]. At the molecular scale, the charge of the chitosan molecule is pH-dependent, with lowering the pH gradually increasing the positive charge due to the protonation of the amine functional groups [24,25,26]. Chitosan is fully or partially positively charged at pH 3, 5, and 7, respectively. Attractive electrostatic interactions between opposite charges result in effective adsorption of chitosan onto silica nanoparticles at low pH conditions. However, as the pH increases, a significant conformational alteration occurs in chitosan solutions, with the zeta potential decreasing. When the pH is 10, chitosan has a weak charge and is not soluble in water, causing significant aggregation of the chitosan binders. Thus, chitosan at pH 10, with a near-zero absolute zeta potential, is not suitable for use in electrode production.

The conformation of binders on silicon particles can be affected by neutralization through various mechanisms. The specific properties of the binder, silicon particles, and neutralizing species present in the solution determine the impact of neutralization on the conformation of binders on silicon particles. One possibility is that the charged species in the solution may interact with the molecules of the binder, altering their conformation [27]. Additionally, changes in the viscosity or other physical characteristics of the solution can affect the conformation of binders on silicon particles. The results from zeta potential analysis indicated that, while binder adsorption occurred across all pH ranges, at pH 10, electrostatic repulsion decreased, leading to poor dispersion of the silicon particles in the solution. This suggests that the zeta potential values can be used as an indicator for understanding the binder adsorption behavior and particle dispersion in a solution. The dispersibility estimated with the zeta potential measurements can be confirmed through examination of the SEM images of the actual electrodes.

**Figure 2:**
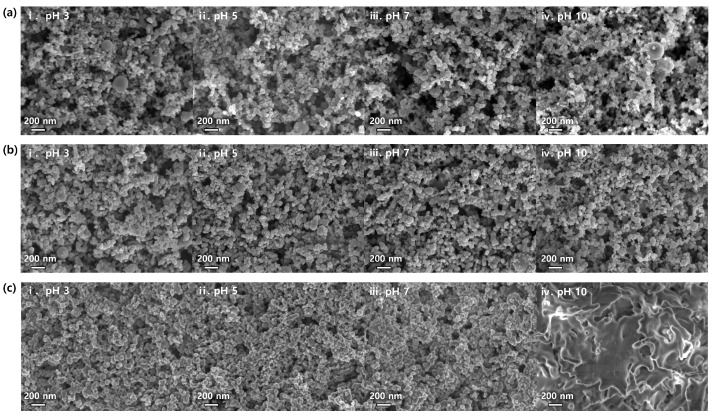
FE-SEM images of anode slurries with various binders at different pH conditions: (**a**) PAA; (**b**) CMC/SBR; (**c**) chitosan: (i) pH 3, (ii) pH 5, (iii) pH 7, and (iv) pH 10.

The stability of the anode particle dispersion is influenced by interactions between the particles and the binder. The morphology of the anode sheets was analyzed using field-emission scanning electron microscopy (FE-SEM). As depicted in Figure 2a–c, the microstructure of the anode sheets with the three different binders under varying pH conditions was investigated. Lower pH levels resulted in more porous structures, leading to distinct particle network arrangements and varying viscosity and viscoelastic properties. However, an aggregation of silicon particles was observed at a pH of 10, attributed to the breakdown of the binder holding the silicon particles and the carbon black together [28]. Among all the electrodes tested under various conditions, the electrode fabricated with the chitosan binder exhibited the most-prominent aggregation at pH 10. At this pH, chitosan exhibited a weak charge and caused the agglomeration of the chitosan binders. The charge of the chitosan molecule at the molecular level is dependent on the pH, with a lower pH gradually increasing the positive charge through protonation of the amine functional groups [24,25,26]. This results in attractive electrostatic interactions between opposite charges and effective adsorption of chitosan onto silica nanoparticles at a low pH. However, the tendency of chitosan binders to aggregate and not adhere to silicon particles makes chitosan unsuitable for use in battery electrodes when the pH is 10 and the absolute zeta potential is close to zero.

**Figure 3:**
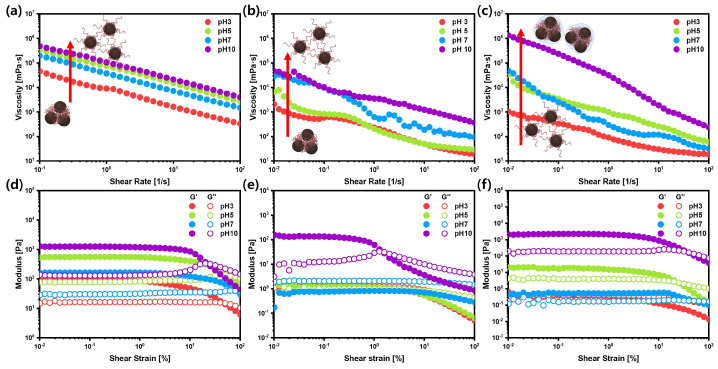
(**a**–**c**) Steady shear viscosity, (**d**–**f**) storage moduli ($G^{\prime }$), and loss moduli ($G^{\prime \prime }$) of silicon anode slurries under various pH conditions with different binders. (**a**,**d**) PAA binder, (**b**,**e**) CMC/SBR binder, and (**c**,**f**) chitosan binder.

The viscosities of silicon anode slurries prepared with various binders were investigated as a function of the shear rate at different pH levels (Figure 3a–c). The viscosity of the anode slurry suspensions was reduced when subjected to shear stress, displaying a shear-thinning characteristic. When a shear stress was applied, the particles underwent deformation and moved relative to each other, leading to a decrease in the effective volume fraction and a reduction in the viscosity of the suspension. It is essential to note that the shear-induced particle rearrangement is only one of the several factors contributing to the observed viscosity decrease. Other factors, such as the break-down of agglomerates, may also play a significant role in reducing the viscosity of the suspension. It was observed that the viscosity of the slurries generally increased as the pH increased, except for those at pH 7. Notably, a significant increase in the viscosity was observed in the slurries prepared with a combination of the PAA and chitosan binders at pH 5. The change in the viscosity of the slurries with the PAA and CMC binders with pH was found to be related to a structural transition caused by the ionization of the carboxylic acid group (RCOOH → H+ + RCOO-). The presence of carboxyl groups in the binder may contribute to the significant increase in the viscosity as a condensation reaction occurs with the surface of the silicon anode particles. The slurry made with uncoiled PAA binders also had a higher viscosity than those made with coiled PAA binders due to increased flow resistance in the solution [29,30]. Chitosan displayed a typical gel-like ($G^{\prime \prime }$ <$G^{\prime }$) behavior at pH 10 due to the neutralization of the amine groups, leading to the elimination of the electrostatic forces between the repulsive chains and the dominance of hydrophobic interactions and hydrogen bonding [31,32,33]. The Si anode slurry made with the CMC/SBR binders at pH 3 to 7 showed a similar behavior to those made with the PAA binders.

The linear viscoelastic properties of the anode slurry were examined using strain sweep tests at various pH levels. The ionization of the carboxylic acid groups in the binder, the presence of carboxyl groups in the binder, the occurrence of a condensation reaction with the surface of the Si anode particles, and the specific properties of the binder were identified as factors that may influence the linear viscoelastic region (LVR) of the slurry. The storage moduli ($G^{\prime }$) and loss moduli ($G^{\prime \prime }$) of the Si-based slurries were measured under strain amplitude sweeps ranging from 0.1 to 100% at a 3 rad/s angular frequency (Figure 3d–f). As the pH of the PAA anode slurry increased, the linear viscoelastic (LVE) range was extended, and $G^{\prime }$ was larger than $G^{\prime \prime }$ throughout the LVR, indicating solid-like behavior in the LVR. An overshoot of $G^{\prime \prime }$ was observed as the pH increased, which represents a structural rearrangement of the Si anode particles coated with the binders, indicating a balance between the formation and destruction of network chains. The slurries with PAA and chitosan binders showed similar trends, with the storage moduli increasing and the LVEs gradually incrementing with increasing pH, suggesting strong binding formation at higher pH levels. The slurries with CMC/SBR binders displayed decreased LVEs as the pH increased, indicating that the network structure of particles in suspensions collapsed at lower shear strains and had worse recovery characteristics.

### 3.2. Binder Adsorptions and Adhesion Strengths

**Figure 4:**
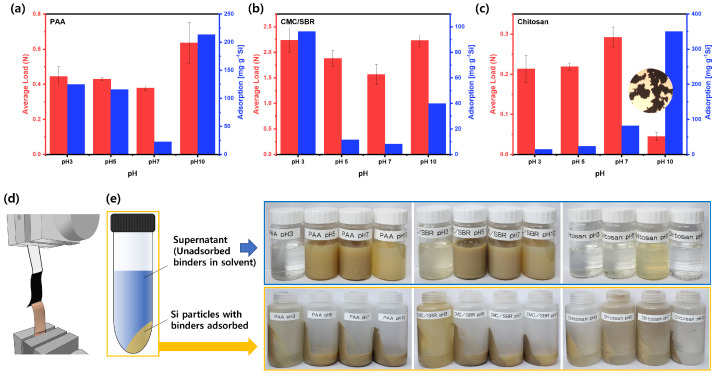
Binder adsorption on Si nanoparticles and adhesion strength of silicon anode slurries with (**a**) PAA binder, (**b**) CMC/SBR binder, and (**c**) chitosan binder on the electrodes. (**d**) Schematic representation of a peeling test and (**e**) silicon anode slurry samples after centrifugation.

The adhesion strength of silicon anode slurries to electrodes is an important aspect that can affect the performance of the electrode [34,35,36]. The binder, which acts as a binding agent for the active material and conductive agent, not only helps to stabilize the electrode material, but also plays a crucial role in improving the lifespan of the electrode and maintaining contact between the slurry and the current collector. To investigate the adsorption of the binders onto the surface of the Si particles, adhesion strength tests were conducted at different pH levels (adsorption in Figure 4a–c). The Si particle sediments with the binders adsorbed were obtained by centrifuging the Si anode slurries, as shown in Figure 4e. The weight loss caused by a temperature increase to 600 ${}^{\circ }$C by TGA was used to calculate the amount of binders that had been adsorbed. The amount of unadsorbed binders present in the supernatant medium varied with the pH. These variations in the amount of unadsorbed binders may impact the overall adsorption of the binders onto the Si particles and the resultant adhesion strength of the coated slurries. Binders containing carboxylate groups can form chemical bonds with Si particles through reactions between the carboxyl group of the binder and the hydroxyl group on the surface of the Si. It was found that the PAA bound more to the Si particles than the CMC/SBR. For both binders, the adsorption decreased from pH 3 to pH 7 and then increased again at pH 10. This is likely due to changes in the conformation of the polymer chains, which can transition from a dense, globular conformation to extended coils. As the pH of the slurry increases, the polymer chains become more negatively charged, which is consistent with the zeta potential results shown in Figure 2. It has been reported that fully extended polymer chains adsorbed on Si nanoparticles can provide steric stabilization, which is more effective than coiled polymer chains [37,38]. Additionally, at a higher pH, the extended conformation of the polymer chains leads to higher surface occupancy, resulting in reduced adsorption of the binders. The adsorption of chitosan binders onto the surface of Si particles is driven by electrostatic interactions between the positively charged chitosan and the negatively charged Si particles. However, at pH 10, a strong aggregation of chitosan binders with each other was observed, as shown in the optical microscope image, which may negatively impact the adhesion force between the Si particles and the chitosan binders. At this high pH, the amino groups in the chitosan binders are not protonated and are unable to form strong electrostatic interactions with the Si particles [26].

In order to gain a comprehensive understanding of the relationship between the adhesive strength of the slurry and the adsorption of binders onto the anode electrode, it is crucial to investigate the mechanism by which the binder and active material interact. The formation of a film or layer on the surface of the active material by the binder can greatly contribute to the overall binding strength of the electrode. The properties of this film, such as the thickness and mechanical properties, can be influenced by various factors such as the type and concentration of the binder, the surface chemistry of the active material, and the processing conditions used to prepare the electrode. A thorough understanding of these factors can aid in optimizing the binder selection and processing conditions to achieve the desired binding strength and performance of the electrode. To quantify the adsorption of binders onto the anode electrode and the resulting binding strength, the adhesive strength of coated Si anode slurries was measured (average loads in Figure 4a–c). The adhesion strengths were measured using a peeling test, as illustrated in Figure 4d. It was observed that the average load was in good correlation with the amount of adsorbed binders, with the exception of the chitosan binder at pH 10. This deviation was likely due to the self-aggregation of the chitosan binders at this pH. These results indicated that the adhesive strength of the slurry with the electrode sheet and the adsorption characteristics of the binder are closely related and can be used to predict the characteristics of the electrode.

### 3.3. Structural Deformation and Recovery of Si Anode Slurries upon Coating Process

The 3ITTs were used to evaluate the effects of shear stress on the deformation and regeneration of the recoverable materials of the Si anode slurries with the various binders. The test provided insights into the rheological behavior of the slurry under different shear rates and pH conditions and how these properties can affect the deformation and recovery of the anode material over time [39,40]. The measurement process was divided into three sections, allowing us to determine the time-dependent changes in the structure of the slurry (SI Figure S2). The first regime represents the idle state before the sample is processed, characterized by a slow mixing rate in the container and a low shear rate. The second regime simulates the high shear that occurs during the application, such as when the slurry is being cast onto a copper current collector. The final regime describes the recovery of the structure after the application has been completed. During the first and third intervals, a low shear stress of 0.1 Pa was applied, and during the second interval, a high shear stress of 100 Pa was applied. The storage moduli and tanδ under the three stress intervals were used to calculate the structural deformation and time-dependent structural recovery parameters. The 3ITTs were performed with a slurry suspension prepared for coating. The calculation method and the physical interpretations are summarized in Table 1. It is crucial to identify appropriate coating conditions for coating the Si anode slurries with different binders to optimize the battery performance while preventing the excessive flow and collapse of the slurry structures.

**Figure 5:**
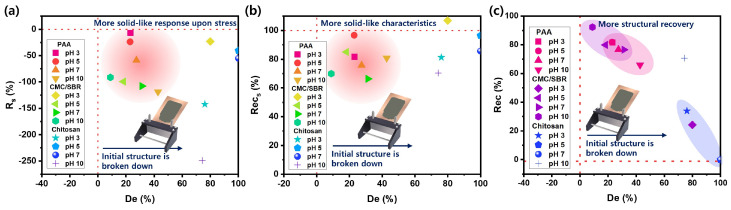
Phase diagrams of (**a**) De—$R_{s}$, (**b**) De—$Rec_{s}$, and (**c**) De—Rec for various binders with Si-based anode slurries.

The phase diagrams in Figure 5 provide a means of evaluating the efficiency of coating on Si anode slurries in relation to their structural deformation characteristics during three-interval thixotropic tests (3ITTs). The deformation factor, De, was used as an indicator of the initial structure’s failure, and when it is close to zero, the material is found to be more resistant to deformation and tends to maintain its current shape. Our research revealed that the Si anode slurries exhibit varying initial break-up, deformation, and recovery characteristics of their structures depending on the pH conditions. Therefore, it is important to control the pH, not only for binder adsorption, but also to improve the efficiency of the slurry coating on the electrode. All of the slurries tested showed positive values of De, negative values of $R_{s}$, and Rec values that were less than 100%. This demonstrated that the slurries exhibit both liquid and partially solid properties with partial recovery, even though their initial network structure has collapsed during the coating process under which the electrode coating process becomes easy. However, it is crucial to identify a suitable coating property so that the structure does not collapse excessively while also not flowing excessively in order to obtain a good performance of the electrode. A significant change in the Rs and Recs values indicates enhanced solid-like characteristics, revealing a highly sensitive flow behavior with respect to the applied shear stress and a low colloidal stability [42,43]. Based on the Rs and Rec vs. De phase diagrams, the optimal coating conditions for the Si anodes slurries with the PAA and CMC/SBR binders were found to occur at pH 3, 5, and 7. The Si anode slurry with the chitosan binder displayed low Rec and high De values, which may not be desirable for some applications, and also showed a low ability to recover from deformation and a low stiffness, which may be detrimental to the coating process.

## 4. Conclusions

We investigated the impact of the surface chemistry on the rheological properties of a slurry of Si anode particles, with a focus on the use of various binders (PAA, CMC/SBR, and chitosan) to prevent particle aggregation and improve flowability and homogeneity. The zeta potential analysis was employed to examine the electrostatic stability of the Si particles in the presence of the different binders. The results indicated that the conformation of the binders on the Si particles can be influenced by both neutralization and pH conditions. Additionally, it was found that the zeta potential values can serve as a useful indicator for assessing binder adsorption and particle dispersion in the solution. Through the analysis of the three-interval thixotropic tests (3ITTs), we discovered that the storage moduli, deformation factor, and recovery coefficient of the slurry varied as a function of the strain intervals, pH conditions, and the chosen binder. The best coating results were observed at pH 3, 5, and 7 with the PAA and CMC/SBR binders. This study highlighted the need to carefully consider the role of surface chemistry, neutralization, and pH conditions in the rheological properties of the slurry and coating quality for lithium-ion batteries.

## Figures and Tables

**Table 1:** Structural deformation parameters obtained from three-interval thixotropic tests (3ITTs) by varying the shear stresses applied to the Si anode slurries [39,41].

Structural Deformation Parameters	Equation	Definition
De (%)	$\frac{G^{\prime }\;\mathrm{at}\;1\mathrm{st}\;3\mathrm{ITT}\;-\;G^{\prime }2}{G^{\prime }1\;\mathrm{at}\;1\mathrm{st}\;3\mathrm{ITT}}\times 100$	Degree of structural deformation (+) De: initial structure is broken down (−) De: further structure is newly developed
Rec (%)	$\frac{G^{\prime }3}{G^{\prime }1\;\mathrm{at}\;1\mathrm{st}\;3\mathrm{ITT}}\times 100$	Degree of structural recovery Rec < 100%: structure is partially recovered Rec > 100%: new structure is generated
$R_{S}$ (%)	$\frac{T1\;\mathrm{at}\;1\mathrm{st}\;3\mathrm{ITT}\;-\;T2}{T1\;\mathrm{at}\;1\mathrm{st}\;3\mathrm{ITT}}\times 100$	Relative solidity (+) $R_{S}$ (%): enhanced solid-like character by stress (−) $R_{S}$ (%): enhanced liquid-like character by stress
$Rec_{s}$ (%)	$\frac{T1\;\mathrm{at}\;1\mathrm{st}\;3\mathrm{ITT}}{T3}\times 100$	Degree of recovery for relative solidity $Rec_{s}$ (%) < 100%: solid character is partially recovered $Rec_{s}$ (%) > 100%: more solid-like character

## Data Availability

Not applicable.

## Supplementary Materials

The following are available online at https://www.mdpi.com/article/10.3390/polym15051152/s1, Figure S1: Diagram illustrating the steps involved in the preparation of silicon anode slurries for a coating process. Figure S2: Three-interval thixotropic tests (3ITTs) and tanδ values of Si anode slurries with various binders.
