# Supplementary material for: Dispersion Homogeneity of Silicon Anode Slurries with Various Binders for Li-Ion Battery Anode Coating
Source: Polymers (Basel). 2023 Feb 24;15(5):1152. doi: 10.3390/polym15051152 (PMC10007508; doi:10.3390/polym15051152)
Supplement: Supplementary file 1 [file polymers-15-01152-s001.zip › polymers-2202597-supplementary.pdf]

Supporting Information

Dispersion homogeneity of silicon anode slurries with various binders for Li-ion battery anode coating

Bogyoung Kim, Yeeun Song, Byungwook Youn and Doojin Lee

Department of Polymer Science and Engineering, Chonnam National University, Gwangju 61186, South Korea.

Preparation of Si anode slurries:

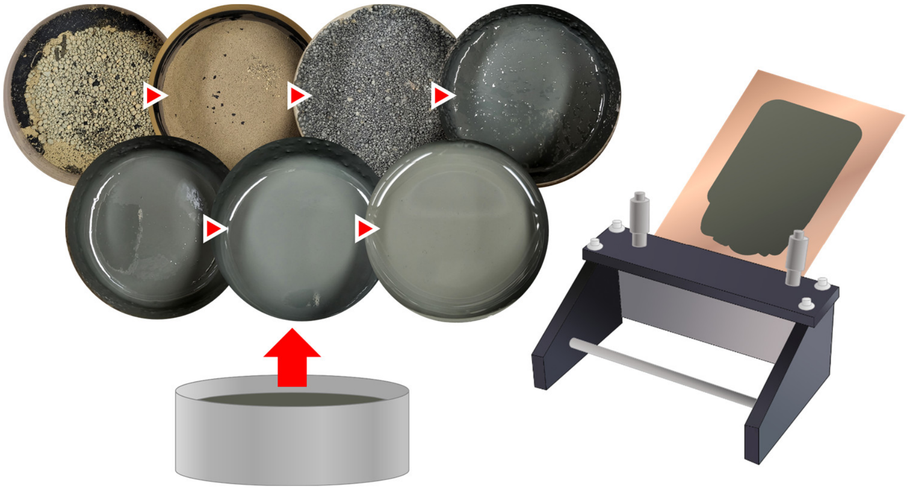

SI Figure S1. A mixing step of silicon anode suspensions used before a coating process: the active material (Si), conductive additive (CB), and binding agents (PAA or CMC/SBR or Chitosan) are mixed in DI water using a planetary centrifugal mixer, and then 1 ml of solvent (DI water) is added per 0.1 g of the total particle.

Results of Three interval thixotropic tests (3ITT):

The 3ITT test was used to investigate how shear stress affects the deformation and recovery of Si anode slurry materials with different binders. The 3ITT values were used to calculate the structural deformation parameters. The measurement process is divided into three sections, allowing us to determine the time-dependent changes in the structure of the slurry (SI Fig. 2). The first regime represents the idle state before the sample is processed, characterized by a slow mixing rate in the container, and low shear rate. The second regime simulates the high shear that occurs during the application, such as when the slurry is being cast onto a copper current collector. The final regime describes the recovery of the structure after the application has been completed. During the first and third intervals, a low shear stress of 0.1 Pa was applied and during the second interval, a high shear stress of 100 Pa was applied. The storage modulus and $\tan\delta$ under the three stress intervals were used to calculate the structural deformation and time-dependent structural recovery parameters (Fig. 5 in the main text).

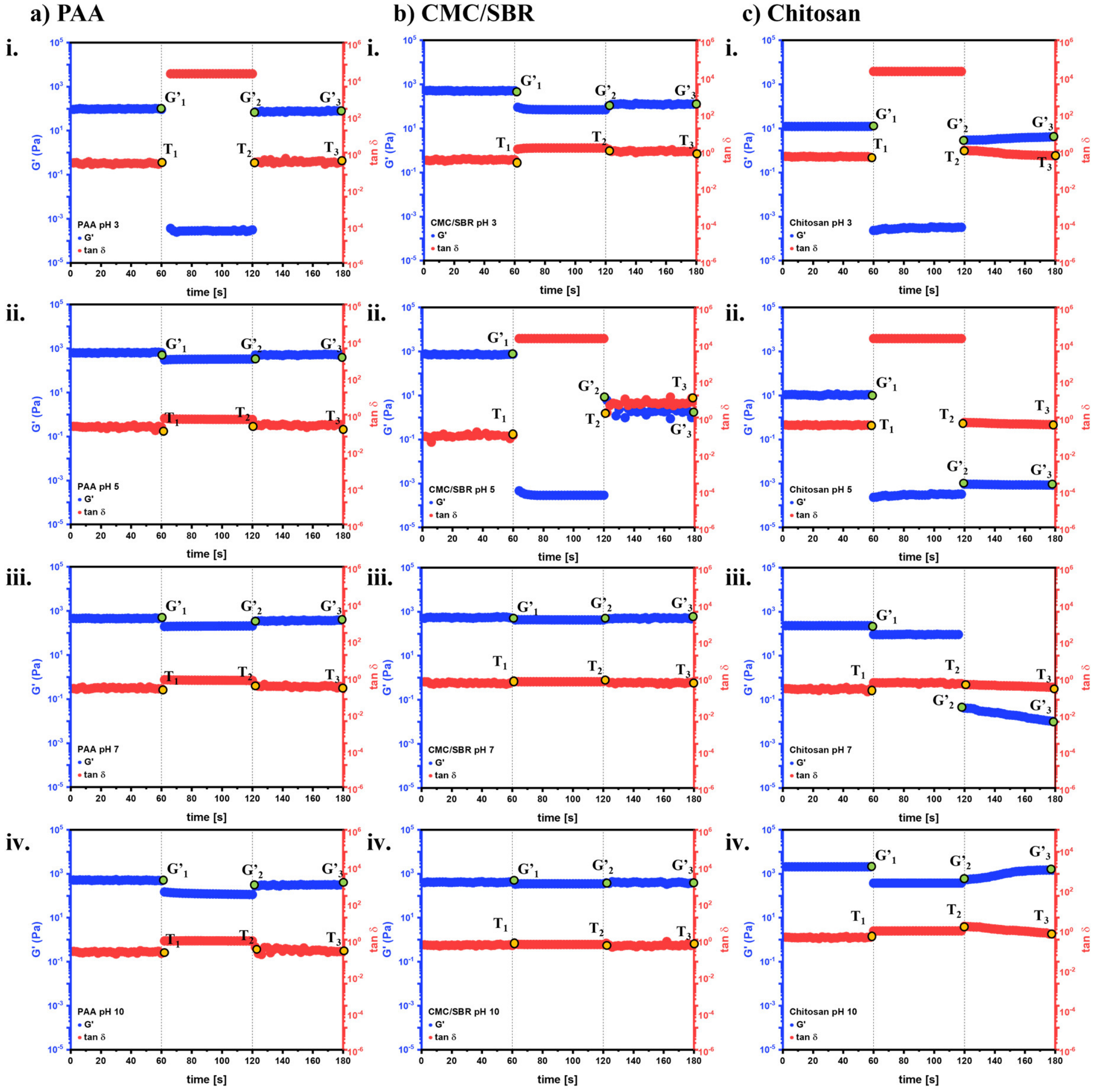

SI Figure S2. Three-interval thixotropic test results of silicon anode slurries with PAA, CMC/SBR, and chitosan binders at different pH conditions: a) PAA binder, b) CMC/SBR binder, and c) chitosan binder at i. pH 3, ii. pH 5, iii. pH 7, and iv. pH 10.
